# Virus-like Particles and Spectral Flow Cytometry for Identification of Dengue Virus-Specific B Cells in Mice and Humans

**DOI:** 10.3390/v18010058

**Published:** 2025-12-30

**Authors:** Katherine Segura, Fabiola Martel, Manuel A. Franco, Federico Perdomo-Celis, Carlos F. Narváez

**Affiliations:** 1 Instituto de Genética Humana, Facultad de Medicina, Pontificia Universidad Javeriana, Bogotá 110231, Colombia; angie_segura@javeriana.edu.co (K.S.); tfabiolacardona@javeriana.edu.co (F.M.); mafranco@javeriana.edu.co (M.A.F.); perdomo_federico@javeriana.edu.co (F.P.-C.); 2 División de Inmunología, Programa de Medicina, Facultad de Ciencias de la Salud, Universidad Surcolombiana, Neiva 410010, Huila, Colombia

**Keywords:** B cells, plasmablasts, dengue, flow cytometry, virus-like particles

## Abstract

Severe dengue virus (DENV) infections are associated with circulating non-neutralizing antibodies generated during heterotypic infections. Although antibodies are key mediators of both protection and pathogenesis, the specific dynamics of B cells (Bc) and their antibody responses remain insufficiently characterized due to limited methods of identifying DENV-specific Bc (DENV-Bc) and the absence of animal models resembling the human disease. Here, we developed a spectral flow cytometry assay employing biotinylated virus-like particles (VLPs) to detect DENV-Bc in C57BL/6 mice and children hospitalized with dengue. DENV-1 and DENV-2 VLPs were biotinylated, and the efficiency of biotin incorporation was assessed with an HABA-avidin assay and ELISA. Serotype specificity and optimal binding conditions were confirmed using hybridomas 4G2 (pan-flavivirus) and 3H5-1 (DENV-2 specific). Fluorescent agglutimers were subsequently generated by coupling biotinylated VLPs to streptavidin–fluorochrome complexes. Splenocytes from intraperitoneally DENV-infected mice and peripheral blood mononuclear cells (PBMCs) from naturally infected pediatric patients were stained with these VLPs and Bc-lineage markers. Biotinylated VLPs bound specifically to hybridomas, and this binding was competitively inhibited by unlabeled VLPs. After secondary DENV challenge, VLPs identified DENV-specific class-switched plasmablasts in mice. Circulating DENV-specific plasmablasts were also detected in children, with agglutimers enabling the discrimination of serotype-specific and cross-reactive responses in primary and secondary infections. This VLP-based approach represents a scalable platform to investigate the protective and pathogenic roles of DENV-Bc in infection and vaccination.

## 1. Introduction

Dengue is a major arthropod-borne viral disease caused by infection with one of the four serotypes of dengue virus (DENV 1–4) [1]. In 2024, more than 12 million suspected dengue cases were reported across the Americas [2]. Despite this substantial disease burden, the development of effective antiviral therapies and a vaccine providing broad, long-lasting protection across all serotypes and demographic groups continues to pose a major challenge [1,2].

B cells (Bc) and antibody responses are central mediators of both protective and pathogenic immune responses against DENV [3,4,5,6]. Severe disease outcomes are typically driven by non-neutralizing or sub-neutralizing antibodies targeting the viral envelope (E) or pre-membrane (pr-M) proteins during heterotypic secondary infection, facilitating via viral entry into Fc receptor-bearing cells through a mechanism known as antibody-dependent enhancement (ADE) [1,4]. Conversely, the production of long-lived neutralizing antibodies confers protection against symptomatic infections [7,8]. This protective antibody response arises from a robust Bc activation, characterized by a massive and transient expansion of plasmablasts (PB) during the acute phase of both primary and secondary infections. The magnitude of this PB burst is notably higher in secondary and severe cases [9,10,11,12]. Studies using ELISPOT and monoclonal antibodies (mAb) generation from dengue patient-derived PB [3] have demonstrated that DENV-Bc responses are largely serotype-specific in primary infection [13,14] and predominantly cross-reactive during secondary infection, likely contributing to the increased severity of secondary infections [3,11,12,13,14,15].

Despite their crucial role, the complete mechanisms by which DENV-Bc and their antibodies contribute to the pathophysiology of dengue remain unclear. This knowledge gap is partially because of the lack of animal models that faithfully reproduce the virological, immunological, and clinical features of human dengue [16,17]. Though wild-type immunocompetent mice are naturally resistant to DENV infection, the mouse models have nevertheless been instrumental in elucidating fundamental aspects of dengue research. Traditional models of primary and secondary infection have typically employed immunocompromised mice lacking IFN receptors or STAT signaling [17,18]. In particular, the AG129 model, deficient in both IFN-α/β and IFN-γ receptors, has been extensively used in recent years, yielding valuable insights into DENV pathogenesis, such as demonstrating that ADE can occur in vivo, facilitated by pre-existing and maternally transferred antibodies [19,20]. Nonetheless, the difficulty in extrapolating the disease mechanisms found in immunodeficient mice to those in immunocompetent hosts is the most significant limitation of this model. More recently, an immunocompetent C57BL/6 mouse model was used to study heterotypic secondary infection using non-mouse-adapted DENV strains to evaluate the role of adaptive immunity in the pathogenesis of dengue [21]. This model may be useful for studying the physiological immune response to DENV.

A further limitation is the difficulty developing methods and reagents to identify and characterize antigen-specific Bc. Virus-like particles (VLPs), which structurally resemble native virions but lack genetic material, have been explored as vaccine candidates [22]. Previous studies successfully employed fluorescent or biotinylated rotavirus VLPs to identify and sort virus-specific Bc by flow cytometry in mice [23] and humans [24]. Similarly, fluorescent bacteriophage Qβ VLPs have enabled the characterization of specific splenic Bc in immunized mice [25]. However, this VLP-based detection strategy has not previously been applied to identify mouse and human DENV-Bc.

To overcome these limitations, in this study, we developed and standardized a biotinylated VLP-based flow cytometry assay to characterize DENV-Bc in an immunocompetent murine model and hospitalized children. Our findings show that this approach constitutes a useful tool with potential applications in studies of pathogenesis and vaccine efficiency evaluation.

## 2. Materials and Methods

### 2.1. Ethics Statement

This study was approved by the ethics committee of the Facultad de Medicina, Pontificia Universidad Javeriana (Minute No. FM-CIE-0781-19), and by the ethics committee of the Hospital Universitario Hernando Moncaleano Perdomo (Minute No. 02-07-2023), in compliance with the Declaration of Helsinki. Written informed consent was signed by the parents or legal guardians of all participating children.

All procedures with mice were approved by the Institutional Animal Care and Use Committee (IACUC) of Pontificia Universidad Javeriana (FUA N° 135-22), in accordance with international standards of animal care and use accredited by AAALACI (Association for Assessment and Accreditation of Laboratory Animal Care International; approval code N° C-563-22).

### 2.2. Biotinylation and Efficiency of DENV VLPs

DENV-1 and DENV-2 VLPs acquired from Native Antigen Company (Oxford, UK; SKU: DENV1-VLP-500 and DENV2-VLP-500, respectively), comprising prM, membrane, and E proteins, were biotinylated using EZ-Link™ Sulfo-NHS-LC-Biotin kit (Thermo Scientific, Waltham, MA, USA; Ref. 21435), following the manufacturer’s instructions. Briefly, VLPs were incubated with the biotin reagent at the recommended molar ratio for 2 h on ice, and excess of unbound biotin was removed by buffer exchange with Zeba™ Spin Desalting Column (Thermo Scientific; Ref. 89877). Protein concentrations were measured using a Nanodrop™ 2000 spectrophotometer (Thermo Scientific) before and after the procedure to ensure sample integrity.

Two complementary approaches verified the biotin incorporation. First, the number of biotin molecules per mole of protein was quantified using the HABA-avidin displacement assay, following the manufacturer’s guidelines (included in EZ-Link™ Sulfo-NHS-LC-Biotin kit). Second, an ELISA-based assay was conducted by coating plates overnight at 4 °C with anti-flavivirus E protein mAb 4G2 (Novus Biologicals, Centennial, CO, USA; Ref. NBP2-52709). After washing and blocking, serial dilutions (1.0, 0.5, 0.25, and 0.125 μg/mL) of biotinylated VLPs were added and binding was detected with 0.5 µg/mL streptavidin-horseradish peroxidase (Kirkegaard & Perry Laboratories, Gaithersburg, MD, USA; Ref: 14-30-00), followed by Tetramethylbenzidine (Kirkegaard & Perry Laboratories, Gaithersburg, MD, USA; Ref: 50-73-03) and measurement at 450 nm. Non-biotinylated VLPs (1.0 μg/mL) served as a negative control. In pilot studies, the optimal working concentration of VLPs was determined.

### 2.3. VLP Binding Assay to Hybridomas

To confirm successful VLP biotinylation, evaluate serotype cross-reactivity, and define the optimal working concentration, two hybridoma cell lines were employed: D1-4G2-4-15 (ATCC HB-112, pan-flavivirus specific, hereafter referred to as 4G2) and 3H5-1 (ATCC HB-46, DENV-2-specific). Serial dilutions of DENV-1 and DENV-2 VLPs (0.5, 1.0, 2.0, 4.0, and 8.0 µg/test) were incubated with the hybridomas, and binding was detected using 0.06 µg/test of streptavidin-PE (BioLegend, San Diego, CA, USA; Ref: 405204). Flow cytometric data were collected on BD FACSCanto II or Cytek Aurora flow cytometers and analyzed using Spectroflo 3.3.0 (Cytek Biosciences) and FlowJo v. 10 (Becton Dickinson) software.

An additional competition test to assess type-specificity was performed. The 3H5-1 hybridoma was incubated for 20 min at room temperature with non-biotinylated DENV-2 VLPs (2.0 and 8.0 µg/test) or PBS 1X. Subsequently, 2.0 µg/test of biotinylated DENV-2 VLPs were added to 3H5-1 cells. As a positive control, a cross-reactive goat anti-human IgG–Biotin (Southern Biotech, Birmingham, AL, USA; Ref. 2040-08; reported to cross-react with mouse IgG) was included. As a negative control, non-biotinylated DENV-1 or DENV-2 VLPs were used. The Binding of VLPs and the positive control was detected with streptavidin-PE, and data were acquired and analyzed as described above. Binding inhibition was quantified by comparing mean fluorescence intensity (MFI) with and without competition.

### 2.4. Mouse Infection and Splenocyte Isolation

**Figure A1:**
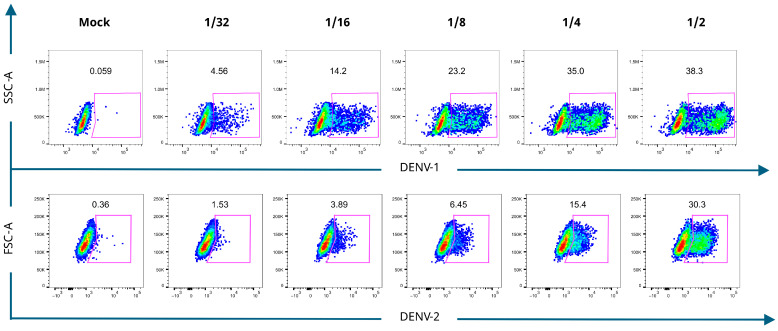
PR/94 and DENV-2 Tonga/74 stocks inoculated into mice.

We used a previously described immunocompetent mouse model of DENV infection [21]. Five-to-seven-week-old female and male C57BL/6 mice (Charles River Laboratories, Wilmington, MA, USA) were inoculated intraperitoneally (i.p.) with 3.0 × 10^8^ Genome Equivalents (GE [26]; obtained by one-step RT-qPCR [27]) of DENV-1 PR/94, DENV-2 Tonga/74 [21]. Viral stocks were kindly supplied by Dr. Steve Whitehead from the Laboratory of Infectious Diseases, NIAD, NIH, DHHS. Mock-infected cell supernatants served as a negative control. Mice were housed under specific pathogen-free conditions in individually ventilated cages at the Comparative Biology Unit of the Pontificia Universidad Javeriana. At fifty-six days post-infection [21], animals were challenged i.p. with 1.0–2.0 × 10^6^ GE of DENV-2 Tonga/74 or mock supernatant. Viral infectivity was additionally evaluated by detecting DENV-1 or DENV-2 infected U937-DC-SIGN cells (ATCC; Ref: CRL-3253) using flow cytometry (Appendix A, Figure A1). Seven days after the challenge, mice were euthanized, and spleens were collected. Spleens were aseptically macerated and homogenized using a syringe and pipetting. Tissue fragments were removed, red blood cells were lysed using RBC Lysis buffer 1X (BioLegend, San Diego, CA, USA; Ref. 420301) for 5 min at room temperature. The resulting splenocytes were filtered and counted using exclusion staining with trypan blue (BIO-RAD, Hercules, CA, USA; Ref: 145–0013).

### 2.5. Flow Cytometry Staining for Mouse Splenocytes

Approximately 2 million freshly isolated splenocytes were stained per test with an 8-color antibody panel consisting of the cell viability Zombie Aqua (BioLegend, Ref. 423102), and the following anti-mouse antibodies: anti-B220 APC-Fire 750 (clone RA3-6B2; BioLegend, Ref. 103259), anti-IgM PE/Cy7 (clone RMM-1; BioLegend, Ref. 406514), anti-GL7 PerCP-Cy5.5 (clone GL7; BioLegend, Ref. 144610), anti-CD19 BV570 (clone 6D5; BioLegend, Ref. 115535), anti-CD138 BV785 (clone 281-2; BioLegend, Ref. 142534), anti-CD3 BV711 (clone 17A2; BioLegend, Ref. 100241), and anti-IgD APC (clone 11-26c.2a; BioLegend, Ref. 405714). DENV-Bc were identified using biotinylated DENV-1 or DENV-2 VLPs at optimized concentrations, followed by streptavidin-PE. Between 1 and 2 million events per sample were acquired on a spectral Cytek Aurora flow cytometer, and the data were analyzed using Spectroflo and FlowJo v. 10 software.

### 2.6. Agglutimer Formation

Agglutimers are multivalent assemblies generated by independent combining biotinylated VLPs from each DENV serotype with streptavidin molecules conjugated to different fluorochromes. Agglutimers of biotinylated DENV-1 and DENV-2 VLPs were generated by incubating VLPs with streptavidin-FITC (BioLegend; Ref. 405202) and streptavidin-PE, respectively, at a concentration ratio of 3:1 (VLP: streptavidin) for 30 min at room temperature protected from light [28]. To remove the unbound fluorochrome conjugates, mixtures were dialyzed using ultrafiltration discs with a molecular weight cut-off of 500 kDa (Amicon, Burlington, MA, USA; Ref. PBVK04310). The purified DENV-1–FITC and DENV-2–PE agglutimers were stored at 4 °C until use in subsequent flow cytometry assays.

### 2.7. Agglutimer-Binding Assays to Hybridomas

To further evaluate the functionality and reactivity of the agglutimers, a binding assay with hybridomas 4G2 and 3H5-1 was performed. Approximately 5.0 × 10^5^ cells per test were incubated with 2.0 µg/test of DENV-1-FITC and/or DENV-2-PE for 30 min at 4 °C, protected from light. Upon washing, cells were acquired on a BD FACSCanto II or Beckman Coulter DxFLEX flow cytometers.

### 2.8. Detection of DENV-Bc in Children Using Individual VLPs and Agglutimers

PBMCs were obtained from pediatric patients with a confirmed diagnosis of dengue with warning signs (DWS) or severe dengue (SD), according to the revised WHO 2009 guidelines [29], who were hospitalized at the Hospital Universitario Hernando Moncaleano Perdomo (Neiva, Huila, Colombia). Cells were isolated by density gradient centrifugation using Ficoll-Paque™ Plus (Cytiva, Darmstadt, Germany; Ref. GE17-1440-02) and either stained immediately or cryopreserved in liquid nitrogen. For flow cytometry staining, the cryopreserved PBMCs were rapidly thawed and washed with RPMI 1640 supplemented with 20% fetal bovine serum, 2mM L-glutamine, 100 U/mL penicillin, and 100 μg/mL streptomycin (all reagents from GIBCO, NYC, NY, USA). A total of 0.5–1.0 × 10^6^ cells per test were stained following a protocol combining surface and intracellular staining. Initially, cells were incubated with Zombie Aqua viability dye for 15 min at room temperature. Subsequently, cells were incubated with the following anti-human antibodies: anti-CD19 BV421 (BD; Ref. 562440) or anti-CD19 V500 (BD; Ref. 561125), anti-CD38 PerCP-Cy5.5 (BD; Ref. 551400), and anti-CD27 PE-Cy7 (BioLegend; Ref. 356412) or anti-CD27 BV421 (BD; Ref. 562513). Cells were then fixed, and permeabilized with Cytofix/Cytoperm (BD, San Jose, CA, USA; Ref: 554722) for 20 min at 4 °C, followed by washing with 1X Perm/Wash buffer (BD; Ref: 554723). For intracellular staining, two strategies were used. In the first, cells were separately incubated with 2 µg/test of either DENV-1 or DENV-2 biotinylated VLPs, followed by streptavidin-PE to reveal binding. In the second, cells were incubated with a mixture of DENV-1–FITC and DENV-2–PE agglutimers (2 µg/test each) to enable the simultaneous detection of serotype-specific or cross-reactive Bc responses. In both cases, incubations were carried out for 20 min at 4 °C, followed by washing. Finally, intracellular immunoglobulins were stained with anti-human IgM APC-Fire 750 (BioLegend; Ref. 314546), and anti-human IgG APC (Jackson ImmunoResearch, West Grove, PA, USA; Ref. NC0100710; diluted 1/50 in FACS buffer containing normal goat serum). Samples were washed, resuspended in Perm/wash 1X, and between 0.5 and 1 million events were acquired on a BD FACSCanto II or Cytek Aurora flow cytometers.

### 2.9. Statistical Analysis

GraphPad Prism 8.0 (GraphPad Software, La Jolla, CA, USA) was used for statistical analysis. Data are presented as median with range. Pearson’s correlation coefficient was used to evaluate linear associations between variables. Comparisons among three or more independent groups were conducted using the Kruskal–Wallis test, followed by Dunn’s multiple comparison test when the Kruskal–Wallis *p*-value was <0.05. Categorical variables were analyzed using the Chi-squared test. Statistical significance was set at *p* < 0.05.

## 3. Results

### 3.1. Characterization of Biotinylated VLPs

Initially, DENV-1 and DENV-2 VLPs were biotinylated using a commercial assay. To rule out protein loss during biotinylation and buffer exchange, VLPs concentration was measured before and after the procedure by spectrophotometry, yielding 0.502 vs. 0.537 mg/mL for DENV-1, and 0.197 mg/mL vs. 0.206 mg/mL for DENV-2 VLPs. Thus, no changes in the protein concentration values were observed after the biotinylation, indicating that the original VLPs content was preserved.

**Figure 1:**
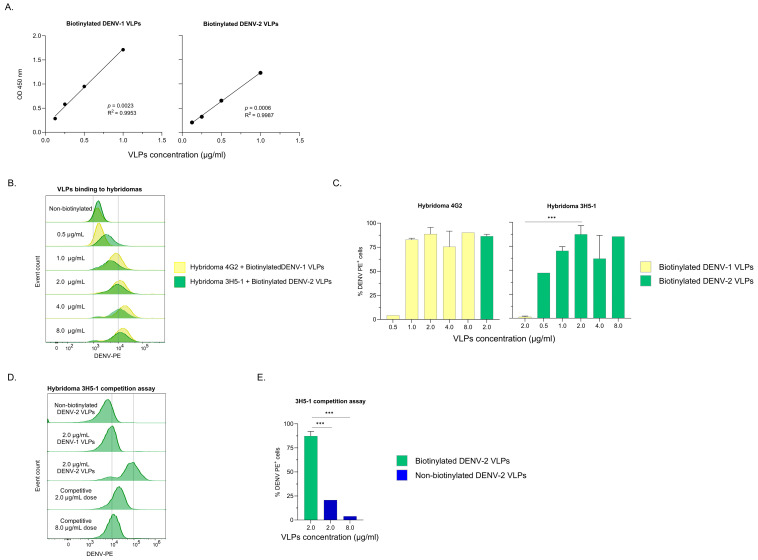
Evaluation of the VLP biotinylation and assessment of its specificity and reactivity in hybridomas. (**A**) Dose-dependent response demonstrates efficient incorporation of biotin into VLPs. Linear regression analysis of the concentration of biotinylated DENV-1 or DENV-2 VLPs analyzed by an ELISA-based method and absorbance detected at 450 nm (**n** = 3 independent experiments per VLP serotype). (**B**) Representative flow cytometry histograms and (**C**) quantification illustrating serotype-specific binding: biotinylated DENV-1 and DENV-2 VLPs selectively bind the pan-flavivirus hybridoma 4G2, while exclusive DENV-2 binding is observed for the serotype-specific 3H5-1 hybridoma (**D**) Histogram plot and (**E**) bar chart of competition assay. “Competitive” condition doses correspond to the concentration of non-biotinylated DENV-2 VLPs shown in the plot, followed by a constant 2.0 µg/mL dose of biotinylated DENV-2 VLPs. Bars represent the median and range from one to five independent experiments. Spearman correlation and Chi-squared tests were applied. *** *p* < 0.0001.

The efficiency of biotinylation was determined by calculating the number of biotin molecules incorporated per mole of protein. Using the Thermo Scientific online HABA calculator [30], incorporation rates were estimated at 2.9 biotin/mole of protein for DENV-1 and 4.1 biotin/mole of protein for DENV-2, an expected value for this procedure [31]. Additionally, an ELISA-based method to detect the biotin bound to VLPs demonstrated a clear dose-effect with a strong linear correlation between the concentration of biotinylated VLPs and the absorbance at 450 nm (DENV-1: *p* = 0.0023, R^2^ = 0.9953; DENV-2: *p* = 0.0006, R^2^ = 0.9987; Figure 1A).

Binding assays with hybridomas demonstrated specific and functional reactivity of biotinylated VLPs. DENV-1 and DENV-2 VLPs bound the pan-flavivirus hybridoma 4G2, whereas the DENV-2–specific 3H5-1 clone bound exclusively to DENV-2 (Figure 1B,C). Consistently, biotinylated DENV-2 VLPs-3H5-1 signal was significantly inhibited by the addition of non-biotinylated DENV-2 VLPs at either equal or four-fold higher concentrations (*p* < 0.0001; Chi-square test; Figure 1D,E), supporting binding specificity and antigenic integrity of the biotinylated VLPs.

### 3.2. Detection of DENV-Bc in Mouse Splenocytes with VLPs

**Figure 2:**
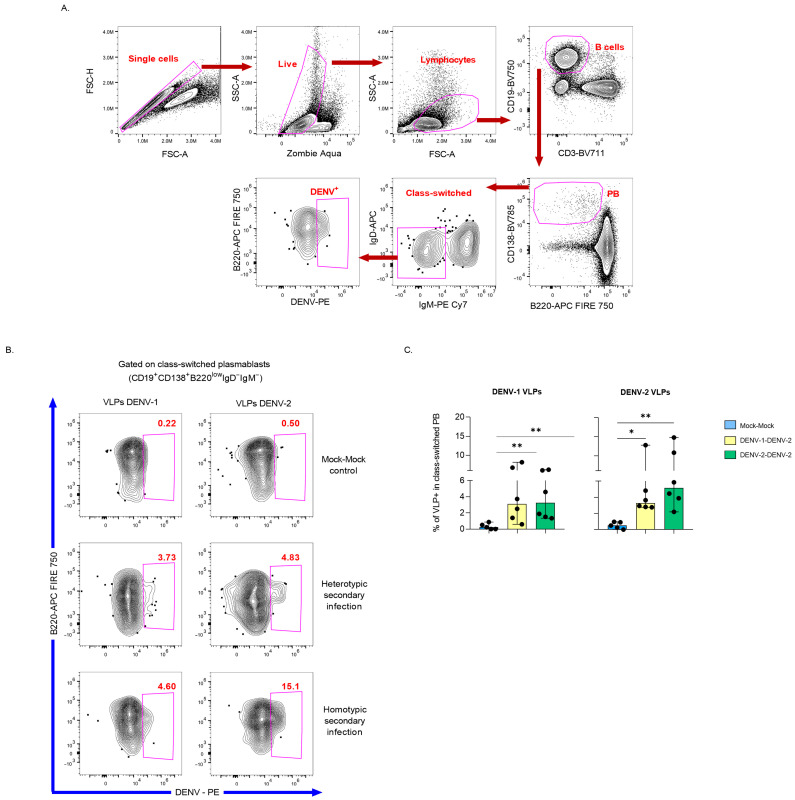
Flow cytometry detection of DENV-specific class-switched PB in mouse splenocytes following DENV infection and challenge. (**A**) Gating strategy to identify DENV-specific class-switched PB (CD19^+^CD138^+^B220^low^IgD^−^IgM^−^) in fresh splenocytes from C57BL/6 mice, exemplified by a homotypically infected individual. (**B**) Representative dot plots showing detection of DENV-specific class-switched PBs in three groups: heterotypically infected (primary DENV-1 followed by secondary DENV-2 challenge), homotypically infected (DENV-2 followed by DENV-2), and negative controls (mock-infected, mock-challenged). (**C**) Summary of DENV-specific class-switched PB frequencies across experimental groups (**n** = 5–6 per group), displayed as median and range. Each black dot denotes one mouse. Statistical analyses used the Kruskal–Wallis test and Dunn’s multiple comparison post-test; * *p* < 0.05, ** *p* < 0.01.

Severe dengue is frequently associated with heterotypic secondary infections, largely due to the generation of non-neutralizing antibodies. Here, we implemented an immunocompetent mouse model of homotypic and heterotypic infection suitable for detecting splenic DENV-Bc [21]. The gating strategy is illustrated in Figure 2A. The PB population was defined as CD138^+^ B220^low^ Bc. Class-switched PB (IgD^−^IgM^−^) were analyzed to identify DENV-specific cells in homotypic and heterotypically infected mice, but they were absent in mock-infected controls (Figure 2B).

### 3.3. Binding of Agglutimers to Hybridomas

**Figure 3:**
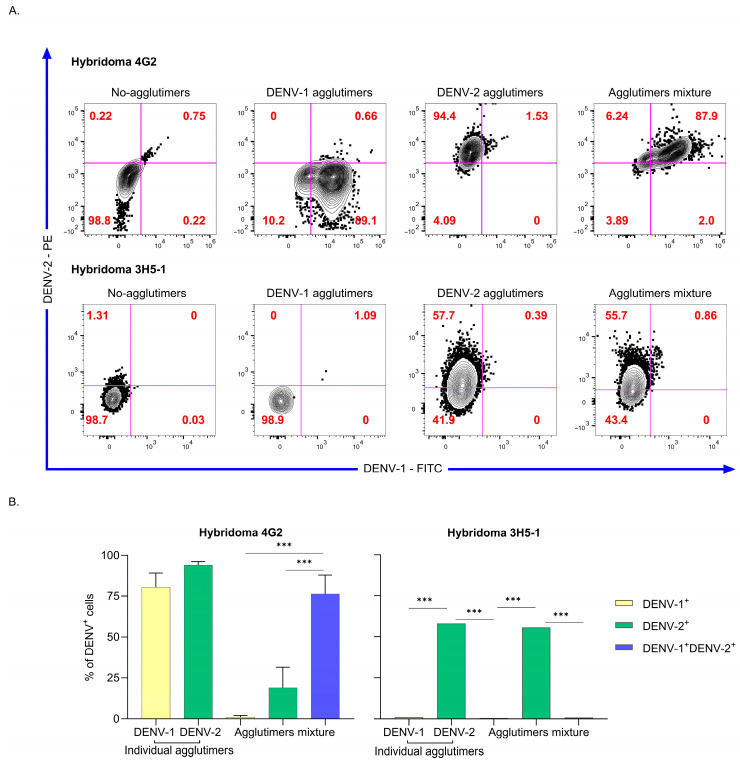
Functional performance of the VLP agglutimers in differentiating serotype-specific and cross-reactive Bc response in hybridoma assays. (**A**) Representative flow cytometry plots illustrate the binding of individual and combined DENV-1 and DENV-2 VLP agglutimers, each labeled with distinct fluorochromes, to two hybridoma cell lines: 4G2 (pan-flavivirus specific) and 3H5-1 (DENV-2 specific). Agglutimers containing both serotypes produce double-positive events in 4G2, indicating cross-reactivity, while 3H5-1 hybridomas bind exclusively to DENV-2 agglutimers, demonstrating specificity and no cross-reactivity. (**B**) Bar graphs summarize quantitative results from two independent experiments, displaying the median and range for the binding frequency of each agglutimer to both hybridoma populations. Statistical analysis was performed using the Chi-squared test (*** *p* < 0.0001).

To simultaneously discriminate serotype-specific and cross-reactive Bc, we generated VLP agglutimers [28]. These agglutimers were generated with biotinylated VLPs labeled with two distinct fluorochromes, and this strategy enables simultaneous binding of VLPS of both DENV serotypes in the same test. The functional evaluation of these agglutimers using hybridomas 4G2 and 3H5-1 confirmed the discriminative capacity. Similar to individual VLPs, both DENV-1 and DENV-2 agglutimers bound to the 4G2 hybridoma, while only DENV-2 bound to 3H5-1 hybridoma when assessed separately (Figure 3A,B). When both agglutimers were combined, the predominant signal in the 4G2 hybridoma corresponded to double positive for DENV-1 and DENV-2 (*p* < 0.0001; Chi-squared). As anticipated, the 3H5-1 hybridoma exhibited binding exclusively to DENV-2 in the agglutimers mixture (*p* < 0.0001; Chi-squared; Figure 3A,B). These results suggest agglutimers can differentiate cross-reactive and serotype-specific responses.

### 3.4. Detection of DENV-Bc in Hospitalized Children with Dengue

**Figure 4:**
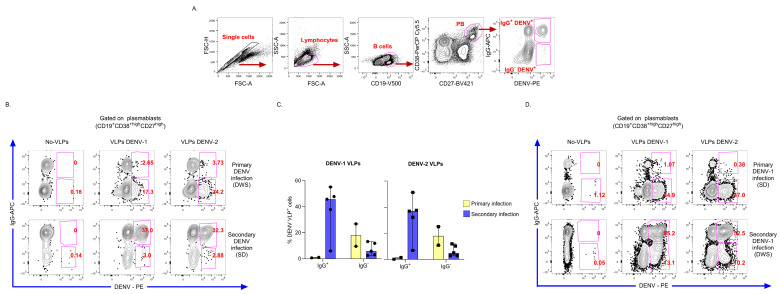
Spectral flow cytometry detection of DENV-PB in PBMCs from hospitalized children with dengue using individual VLPs. (**A**) Gating strategy for identifying DENV-PB in permeabilized PBMCs shown for a representative secondary SD case. (**B**) Illustrative flow cytometry plots depict isotype-specific DENV-PB responses in patients whose infecting DENV serotype was undetermined, including a negative control (no VLPs, streptavidin-PE only) to confirm assay specificity. (**C**) Summary of isotype-specific DENV-PB frequencies in children classified as primary (IgG^−^) or secondary (IgG^+^) infections. Data plotted as median and range, with each dot representing an individual patient. (**D**) Example flow cytometry plots show detection of DENV-PB in patients with confirmed primary or secondary DENV-1 infection, as determined by laboratory testing.

**Figure 5:**
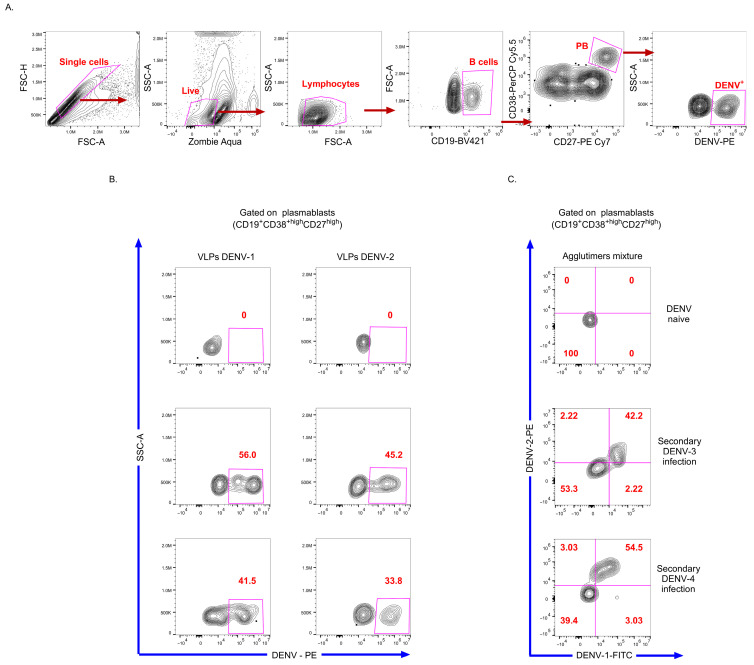
Comparative detection of DENV-PB in PBMCs from hospitalized children with dengue using individual VLPs versus mixed agglutimers. (**A**) Gating strategy for the precise identification of DENV-PB in permeabilized PBMCs from a child with DWS. (**B**) Flow cytometry plots display DENV-PB frequencies detected with single DENV-1 or DENV-2 VLPs in individual patient samples. (**C**) Agglutimer assay results (DENV-1-FITC and DENV-2-PE conjugates) from the same patients demonstrate double-positive cross-reactive DENV-PB compared to single serotype VLPs. PBMCs from a DENV-naive individual serve as a control for assay specificity. Representative data shown exclude two children with DENV-4 infection who have comparable results.

A primary aim of developing the VLP-based assay for detecting DENV-Bc was to demonstrate its clinical applicability in a relevant context and assess its potential to capture ex vivo immune response. After confirming the specificity and performance of the approach in DENV-specific hybridomas and murine splenocytes, we tested the assay with PBMCs from hospitalized children with dengue (demographic, clinical, and laboratory characteristics in Table 1). The gating strategy to identify DENV-PB using individual VLPs or agglutimers is shown in Figure 4A and Figure 5A, respectively. We defined PB as CD38^high^ and CD27^high^ Bc.

We first analyzed the IgG DENV-specific PB response in children with primary or secondary DENV infection, including children in which the serotype of the infecting virus could not be determined (Figure 4B,C) and in which it was determined with RT-qPCR (Figure 4D). Interestingly, in children with primary infections, only a small proportion of IgG^+^ DENV^+^ PB was detected, while most of the DENV-specific response was confined to the IgG^−^ PB (possibly IgM^+^ expressing PB). In contrast, secondary infections were characterized by a markedly higher frequency of IgG^+^ DENV^+^ PB and a lower proportion of IgG^−^ DENV^+^ PB, suggesting the ability of the assay to discriminate isotype-specific DENV-PB responses (Figure 4C). Next, we explored the serotype-specific reactivity of PB in children with RT-qPCR-confirmed DENV-1 or DENV-2 infections. As shown in Figure 4D, comparable frequencies of PB binding to DENV-1 and DENV-2 VLPs were observed in both primary and secondary DENV-1 infections, indicating a highly cross-reactive PB response. This finding supports the notion that early in primary infection, DENV-Bc response is cross-reactive. Additionally, data suggest that secondary dengue is accompanied by a broad activation of mBc cells capable of recognizing conserved epitopes across DENV serotypes [32].

To confirm and extend these findings, we also analyzed the DENV-PB response using DENV-1 and DENV-2 agglutimers in PBMC from children with confirmed secondary DENV-3 and DENV-4 infections. The frequency of PB in the DENV-naive control individual was low, while the frequency of PB in patients with dengue was high (Table 1), in keeping with previous studies [5,9]. Individual biotinylated DENV-1 or DENV-2 VLPs enabled the identification of DENV-PB for both serotypes in the children included (Figure 5B). In addition, the agglutimer mixture revealed a strong double-binding cross-reactive PB response, and only a minor single-binding population of non-cross-reactive PB to DENV-1 or DENV-2 (Figure 5C). No DENV^+^ events were observed in the DENV-naive control, evidencing the specificity of the assay (Figure 5C).

## 4. Discussion

This study describes the development and optimization of a flow cytometry assay design to identify and characterize DENV-Bc in both splenocytes from immunocompetent mice and PBMCs from naturally infected pediatric patients. This assay offers a practical tool for directly assessing Bc responses during DENV infection and vaccination.

Numerous approaches have been employed to investigate DENV-Bc responses, each with inherent advantages and limitations. ELISPOT and FluoroSpot assays have revealed that E protein–specific memory B cells (mBc) are serotype-restricted during the convalescent phase of primary infection but become increasingly cross-reactive following secondary exposure [13,14]. Using purified virions as antigen, these studies showed that the mBc response to the infecting serotype dominates in secondary infections, although cross-reactive responses exceed serotype-specific ones [14]. ELISPOT assays have also enabled the assessment of DENV-specific antibody-secreting cells using complete virions [9,12,15] or recombinant viral proteins [11], providing evidence of a highly cross-reactive response, particularly during the acute phase of the disease. Despite their utility, these techniques require ex vivo cell activation, do not allow phenotypic characterization of the Bc, and cannot reflect direct antigen binding at the single-cell level. Similarly, an approach to studying the DENV-specific Bc involves analyzing serotype-specificity and somatic hypermutation of mAb generated from sorted PB [3]. While informative, this method is labor-intensive and low-throughput. In contrast, our VLP-based assay is direct, rapid, and allows identification of DENV-Bc without requiring stimulation or cloning.

Previous studies have attempted similar strategies using fluorochrome-labeled virions or recombinant viral proteins for direct flow cytometry detection of DENB-Bc [33,34,35]. Alexa Fluor-conjugated virions identified DENV-specific human mBc and PB [33,34], although PB detection remains less clear. However, such methods suffer from variability in virion quality, inefficiency in producing labeled virions for all serotypes, and possible structural alterations induced by direct fluorochrome conjugation. Likewise, fluorochrome recombinant proteins capture only a restricted fraction of the Bc repertoire [35], as several conformational and complex epitopes are not represented [36]. In contrast, our VLP-based method offers uniform structural quality, preserves antigenicity after biotinylation, and captures a broader Bc repertoire [22], making it more versatile and possibly reproducible for routine experimental use.

VLPs biotinylation is a simple and short procedure, whose efficiency can be readily evaluated through standard methods [37]. We standardized and optimized biotinylated VLPs using multiple strategies to ensure reagent efficiency. The dose-effect observed in the ELISA for detecting biotin bound to VLPs reflects the degree of biotin incorporation, as confirmed by the HABA-avidin assay. The pan-flavivirus hybridoma (4G2), which recognizes both DENV-1 and DENV-2 VLPs, and the DENV-2 specific hybridoma (3H5-1) were employed to demonstrate that the biotinylation process preserved the antigenic integrity of the VLPs, and that the biotinylated VLPs bind specifically, consistent with previous reports showing that biotinylated DENV VLPs retain serotype-specific recognition by mAbs [38].

To the best of our knowledge, this study provides the first characterization of DENV-specific splenic Bc using an immunocompetent mouse model. Earlier studies focused on NS3, pr-M, and E-specific B cells in the inguinal lymph nodes of BALB/c mice using individual recombinant antigens and ELISPOT [28,39]. In comparison, our DENV-1 and DENV-2 VLPs successfully detected specific PB in infected mice but not in controls, showing that under the appropriate viral dose and route of administration, this mouse model can generate a Bc response against DENV, and the biotinylated VLPs can evaluate the serotype specificity of this response.

While the agglutimer method was described previously [28], earlier implementations required separate tubes per antigen due to shared fluorochrome labeling, preventing serotype discrimination. In contrast, our VLP agglutimers are used as a mixture for the simultaneous analysis of responses to two DENV serotypes within a single tube. In the hybridoma standardization experiments, most events were double-positive, with a few cells binding only DENV-2 when the 4G2 hybridoma was used, consistent with the fact that the 4G2 clone was originally derived from the DENV-2 New Guinea C strain [40]. Meanwhile, the 3H5-1 hybridoma showed binding only to DENV-2, as expected for a serotype-specific clone.

Individual VLP staining allowed the detection of DENV-1 or DENV-2 specific PB in patients, according to the acute-phase PB expansion previously reported [9,11]. Although mBc were not analyzed here, the method could potentially detect them as well. The preliminary suggested pattern of IgG^−^DENV^+^ PB in primary infections and IgG^+^ DENV^+^ PB in secondary infections could align with previous results of IgM and IgG expressing cells detected by ELISPOT in primary and secondary infections, respectively [11]. The mixture of VLP agglutimers allowed visualization of double-positive cells, indicating a high cross-reactive response even in samples with low cell counts. Single-positive cells may correspond to serotype-specific Bc. No PB binding to VLPs was seen in the DENV-naive individual, demonstrating assay specificity. Importantly, a fraction of samples analyzed had been cryopreserved, yet the method remained effective for detecting DENV-Bc. This suggests that the methodology may be favorable and applicable to longitudinal multicohort patient studies in which cells are frozen.

Several limitations should be acknowledged. The relatively small sample size may limit the generalization of the results; therefore, the human findings presented here should be considered preliminary and require validation in a larger, longitudinal, independent cohort to confirm their robustness and the applicability of this method in diverse epidemiological contexts. We lacked access to DENV-3 and DENV-4 VLPs, which are increasingly prevalent in regions historically dominated by DENV-1 and DENV-2 [41], and thus, results from individuals infected with these serotypes should be interpreted with caution. Additionally, while PB accounts for most of the Bc response during the acute phase, which was the primary focus of this work, a comprehensive understanding of dengue immunity will require extending this approach to mBc, which are essential for long-term protection and vaccine efficacy [42].

In summary, biotinylated VLPs and their agglutimers provide a straightforward, versatile, and scalable tool for evaluating DENV-specific B cells in both animal models and patients. Future extensions could include biotinylation of non-structural proteins such as NS1 to broaden the repertoire of the DENV-Bc for study. Moreover, applying this strategy to other orthoflaviviruses, such as the Zika virus, could provide critical insight into Bc cross-reactivity among related viruses and its impact on disease outcomes and vaccine design.

## Figures and Tables

**Table 1:** Demographic and clinical features of patients included in the DENV-Bc analysis.

	Dengue Naive	Dengue
	(n = 1)	(n = 10)
**Female/Male (n)**	0/1	4/6
**Age (years), median (range)**	4	11 (0.3–17)
**Days of symptoms onset, median (range)**	4	6.5 (5–10)
**Diagnostic test, n (%)**		
ELISA NS1^+^	0	8 (80)
ELISA DENV-IgM^+^	0	8 (80)
ELISA DENV-IgG^+^	0	9 (90)
RT-PCR+, n (%)	0	7 (70)
DENV-1, n (%)	-	2 (20)
DENV-2, n (%)	-	1 (10)
DENV-3, n (%)	-	1 (10)
DENV-4, n (%)	-	3 (30)
**Diagnosis**		
DWS/SD, n (%)	-	6 (60)/4 (40)
**Primary/secondary DENV infection, n (%)**	-	2 (20)/8 (80)
**Frequency (%) of PB in Bc, median (range)**	0.64	9.84 (3.4–32.4)
**Laboratory tests, median (range)**		
Leukocytes (cells/mm^3^)	22,400	5240 (2790–9500)
Hematocrit (%)	29.8	44 (32.4–50.1)
Hemoglobin (mg/dl)	9.5	14.8 (10.4–17.42)
Platelets (cells, mm^3^)	746,000	52,000 (21,000–414,000)
AST (U/L)	15.2	174.9 (31.8–652)
ALT (U/L)	23	121 (14.6–347)

Values are presented as median (range) for continuous variables and relative frequency (%) for categorical variables. A Dengue naive refers to a single child initially suspected of having dengue but later confirmed negative by both serological and molecular diagnostic tests (Including DENV-IgG negative by ELISA). The “Dengue” group comprises all hospitalized children with laboratory-confirmed DENV infection (n = 10). DWS: Dengue warning signs. SD: Severe dengue. PB: Plasmablast. Due to the small size, all results are descriptive, and no inferential statistical comparisons were performed.

## Data Availability

This paper presents all related data and methods. Any additional enquiries should be directed at the corresponding author.

## Abbreviations

The following abbreviations are used in this manuscript:

DENV	Dengue virus
Bc	B cells
DENV-Bc	DENV-specific Bc
VLPs	Virus-like particles
E	Envelope
Pr-M	Pre-membrane
mAb	Monoclonal antibody
PBMCs	Peripheral Blood Mononuclear Cells
DWS	Dengue warning signs
SD	Severe dengue
PB	Plasmablasts
mBc	Memory Bc

## Appendix A

U937-DC-SIGN cells were incubated with 50 μL of dilutions of DENV-1 and DENV-2 stocks from 1/2 to 1/32 for 2 h at 37 °C and 5% CO2. Then, cells were centrifuged, the supernatant eliminated and resuspended in culture medium. After 46 and 22 h of incubation for DENV-1 and DENV-2, respectively, cells were washed and stained with 100 ng/test of mAb 4G2, followed by Brilliant Violet 421™ Goat anti-mouse IgG (San Diego, CA, USA; Ref: 405317). Data was acquired on BD FACSCanto II or Cytek Aurora flow cytometers and analyzed using Spectroflo (Cytek Biosciences) and FlowJo v. 10 (BD) software.
